# Health-related quality-of-life outcomes in CNS WHO grade 2 and 3 meningioma: a systematic review

**DOI:** 10.1007/s10143-025-03420-5

**Published:** 2025-02-27

**Authors:** William H. Cook, Fareha Khalil, Conor S. Gillespie, Adel E. Helmy

**Affiliations:** Division of Neurosurgery, Department of Clinical Neurosciences, University of Cambridge, Cambridge, UK

**Keywords:** Health-related quality-of-life, Meningioma, Patient-reported outcomes, Questionnaires

## Abstract

WHO grade 2 and 3 meningioma constitute approximately 20% of all meningioma. The lower incidence of these more aggressive meningiomas has led to under-representation of clinical outcomes in the literature. It is hypothesised that patients with grade 2 or 3 meningiomas are disabled by tumour and treatment morbidity, contributing to lower health-related quality-of-life (HRQoL). A PRISMA-compliant systematic review was conducted (PROSPERO CRD42023441009). MEDLINE, EMBASE, and Cochrane Library databases were searched between inception and September 2023. Studies of adults (> 16 y.o.) with histologically-proven WHO grade 2 and 3 cranial meningioma who underwent a combination of surgery, radiotherapy, and stereotactic radiosurgery and had HRQoL outcome data were included. Primary outcome was HRQoL. Fifteen studies were included. HRQoL was measured with 10 different tools, three of which have been validated in meningioma patients. Only two studies exclusively reported on WHO grade 2 and 3 meningioma and four further studies considered WHO grade in statistical analysis. WHO grade 2 and 3 meningioma were associated with reduced HRQoL in two studies that reported direct comparison and no difference in another two. Psychological domains were reduced in most studies compared to normative data or controls including in one of the two studies reporting on WHO grade 2 and 3 tumours exclusively. This systematic review highlights the need for prospective studies of more patients with grade 2 and 3 meningioma with validated meningioma-specific HRQoL tools. The current literature is limited by the small proportion of patients within reported studies, and heterogenous and poorly reported management paradigms.

**Supplementary Information:**

The online version contains supplementary material available at 10.1007/s10143-025-03420-5.

## Introduction

Meningioma is the most common primary intracranial tumour [1], with an incidence of 9.5 per 100,000, which increases to 46.8 per 100,000 for patients over the age of 80 [2]. Most meningioma are WHO grade 1, accounting for 80% of cases, with a smaller proportion being WHO grade 2 (atypical) or 3 (malignant) [1]. Patients may present with symptoms such as headache, or signs such as neurological deficit. An increasing number of meningioma are being detected incidentally through diagnostic imaging performed for other purposes [3]. The first line management of symptomatic meningioma is surgical resection [4], with adjuvant therapy offered to those with WHO grade 2 or 3 tumours, residual [5, 6], or recurrent disease [4].

Patients with meningioma have reduced life expectancy compared to the general population, with overall survival rates at 10 years for WHO grade 1, 2, and 3 meningioma being 81%, 63%, and 15%, respectively [7]. Many studies have reported that patients with meningioma have reduced health-related quality-of-life (HRQoL) in comparison to the normative population, across multiple domains such as social functioning, emotional wellbeing, general health, and return to work capabilities following treatment [8]. As incidence and survival increases, understanding the impact of both meningioma occurrence, and impact of treatment, on HRQoL will become increasingly pertinent to both patients and clinicians [9].

Most studies on HRQoL in meningioma include only grade 1 meningioma [10–12], or have reported a combined cohort of WHO grades, consisting of mainly WHO grade 1 meningiomas, reflective of the relative incidence of each meningioma grade. Specific studies of WHO grade 2 and 3 meningioma are less common [13, 14], with previous studies demonstrating increased rates of anxiety and depression, but conflicting evidence regarding HRQoL compared to the general population [15].

It is also unclear if any HRQoL differences exist between WHO grade 2 and 3, and WHO grade 1 meningioma. The latter often follow a different treatment pathway, have lower rates of recurrent disease, and survive longer. A systematic review focussing on WHO grade 2 and 3 meningioma would be helpful in identifying any differences between these groups and ensure healthcare services are responsive to the needs of these patients.

## Methods

### Search strategy

A systematic review was conducted according to the PRISMA guidelines and registered with PROSPERO (CRD42023441009). MEDLINE, EMBASE, and Cochrane Library databases were searched between inception and September 2023 using keywords that were approved by a clinical librarian (Supplementary Materials, Appendix 1). Reference lists of included articles were checked for additional studies. Search terms used included “atypical adj6 meningioma*” and “malignant adj6 meningioma*” and the full Ovid MEDLINE search can be found in Supplementary Materials, Appendix 1, which was adapted for the other electronic databases.

### Paper selection

Studies of adults (> 16 y.o.) with histologically-proven CNS WHO grade 2 and 3 cranial meningiomas who underwent a combination of surgery, radiotherapy, and stereotactic radiosurgery and had health-related quality-of-life (HRQoL) data were included. Chemotherapy trials and trials of other systemic therapies were excluded. Study abstracts, animal studies, reviews, and case reports (up to 5 patients) were excluded. Only papers published in English were included. Two independent reviewers (W.H.C. and F.K.) screened all titles and abstracts for eligibility and categorised studies according to outcome type, in this case patient-reported quality-of-life. Disagreement was resolved with discussion and consensus, and when discussion failed to lead to consensus, a third researcher mediated (C.S.G.).

### Data extraction

Information was extracted from each article by two independent reviewers (W.H.C. and F.K.) using pre-designed forms for study design, main inclusion criteria, subject characteristics (age, sex, CNS WHO grade, tumour location, Simpson grade, adjuvant therapy, and functional status), and study outcomes. The primary outcome of interest for this report was HRQoL. Timing of HRQoL assessment, questionnaire(s) used, and outcomes were extracted. Data are presented for all studies separately. No meta-analysis was performed due to the small number of studies and heterogeneity of the study populations, intervention strategies, and outcomes.

### Assessment of reporting level of patient-reported outcomes in included articles

HRQoL outcome data reporting was assessed by two independent reviewers (W.H.C. and F.K.) following criteria adapted from the International Society of Quality of Life Research (ISOQOL) (Supplementary Materials, Supplementary Table 1) [16]. Eighteen points was the maximum score possible and a score of 13/18 was deemed sufficient reporting in a similar manner to previous publications [17, 18].

**Fig. 1:**
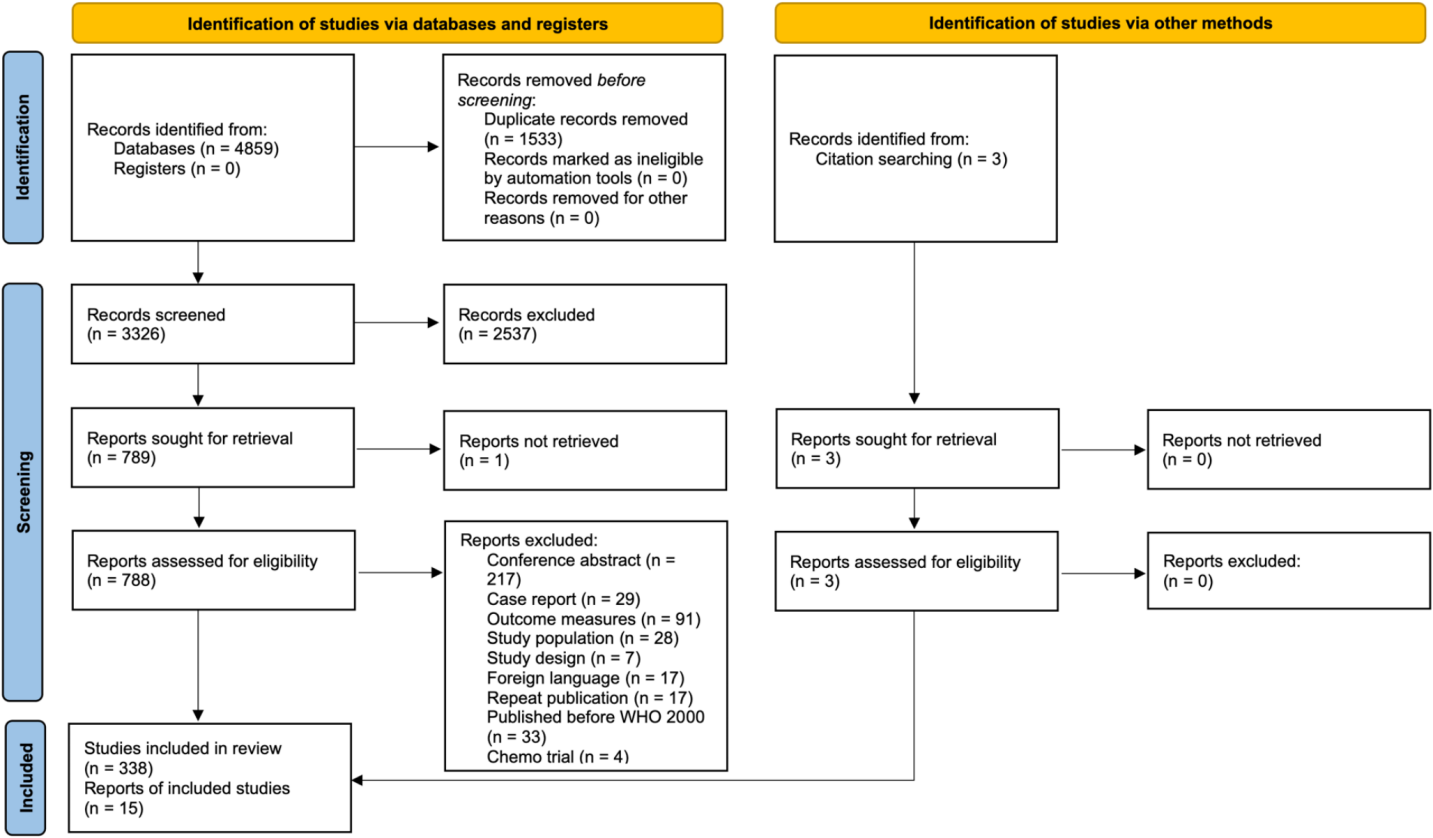
PRISMA 2020 flow diagram of database search, screening, and eligibility assessment for the present study. Eligible studies were categorised according to outcome and 15 studies were identified as reporting HRQoL outcomes

## Results

### Study characteristics

In total, 4859 articles were identified from three electronic databases, 3326 were screened after de-duplication, 770 articles were selected for full-text analysis and 15 were categorised as including HRQoL outcomes, including three studies found through citation searching (Fig. 1; Table 1) [8, 13–15, 19–29]. Of the 15 included articles, five studies were prospective and 10 were cross-sectional. Study population ranged from 45 to 249 patients with all CNS WHO grades of meningioma. Two studies investigated CNS WHO grade 2 and 3 meningiomas only [15, 24], all others included grade 1 meningiomas too. On average, 17% and 6% of meningioma in these studies were WHO grade 2 and 3, respectively. Most studies did not report HRQoL outcomes for CNS WHO grade 2 and 3 tumours separately [8, 13, 14, 19–23, 25]. Four studies included all grades of meningioma and assessed the effect of WHO grade on HRQoL [26–29].

**Table 1:** Summary of included studies reporting on HRQoL outcomes

Author (year)	Study design	Inclusion & exclusion	Patients & controls	Response rate	Age (mean)	Sex (% female)	CNS WHO grade	Location	Simpson grade	Functional status	Primary intervention	HRQoL tools	Moment measured	Main result(s)	Psychology, role functioning
Krcek et al., 2023 [19]	Prospective	Meningioma, pencil beam scanning proton therapy, excluded: no consent, split-course, non-completion, spinal tumours.	200 (83 for QoL). Compared to normative data.	41.5%	50	73%	1: 70%, 2: 28%, 3: 3%	Skull base: 70%, non-skull base: 30%	No resection: 19%, 1-3: 15%, 4-5: 66%	N/A	81% surgery. Proton therapy as initial treatment (56%) or at recurrent/progressive disease (45%).	EORTC QLQ-C30, QLQ-BN20	During treatment (first week, half-way, end) and annually after treatment (median follow-up 65 months).	Proton therapy did not have a negative impact on QoL during follow-up. *CNS WHO grade 2 and 3 tumours not reported separately.*	Medium deterioration cognitive function. Medium improvement in emotional functioning. No change in role function. ~12 point deterioration in future uncertainty.
Kofoed Lauridsen et al., 2023 [27]	Cross-sectional	Histologically confirmed meningioma and brifrontal craniotomy (subfrontal) approach: olfactory groove, planum sphenoidale, tuberculum sellae. Ex: previous cranial surgery, multiple tumours, NF2.	45. Compared to normative and other meningioma and glioblastoma populations.	75%	60	64%	1: 71 (92%), 2: 6 (8%) (of those screened)	100% subfrontal	N/A	N/A	100% surgery	FACT-G, FACT-Br, HADS	Mean 7.1 y postop.	Patients with subfrontal meningiomas had better long-term HRQoL than general populations and other meningioma cohorts. Unclear how many CNS WHO grade 2 tumours included in HRQoL study. WHO grade appeared not to affect FACT-G or -Br scores.	Less anxiety and depression than reference populations. Cognitive function not formally assessed. Role functioning not reported on.
Keshwara et al., 2023 [8]	Cross-sectional	Actively monitored and surgically operated meningioma, ≥16 yo, English-speaking, 5 y of follow-up.	243. Compared to normative data.	N/A	69	80%	1: 85%, 2: 15%	Skull base: 38%, midline: 14%	N/A	WHO Performance Status 0: 45%, 1: 39%, 2: 2: 12%, 3: 2%	64% surgery. 17% radiotherapy.	RAND SF-36, EORTC QLQ-C30, QLQ-BN20	Mean 9.8 y following diagnosis.	Clinically relevant QoL impairments across several domains, similar QoL between actively monitored & operatively managed meningiomas. *CNS WHO grade not included in multivariate analysis as not all had grade data available.*	Worse emotional well-being cf. normative score on SF-36, not significant on QLQ-C30. Worse role functioning due to both physical and emotional problems on SF-36 and QLQ-C30, worse cognition.
Lisowski et al., 2022 [20]	Cross-sectional	Meningioma treated with RT, no spinal infiltration.	119. Compared to normative data.	59.8%	59	64%	None: 36%, 1: 32%, 2: 17%, 3: 15%	Skull base: 61%, falx: 23%, convexity: 13%, optic nerve sheath: 3%	Not known: 6%, 1: 5%, 2: 10%, 3: 2%, 4: 40%, 5: 3%	Median KPS 80 (30-100), KPS ≥90% in 50% and <90% in 50%	69% surgery. Radiotherapy (primary in 31%, adjuvant in 16%, at relapse in 53%).	EORTC QLQ-C30, QLQ-BN20	Median 4.8 y after RT.	Global health status mean 59.9 (QLQ-C30), deterioration of long-term QoL. *CNS WHO grade 2 and 3 tumours not reported separately.*	Decreased role and cognitive function.
Pettersson-Segerlind et al., 2022 [15]	Cross-sectional	Adult *grade 2 and 3 tumours*	51 (18 for QoL). Compared to normative data.	35.3%	64	51%	Overall: 2: 84%, 3: 16%. QoL: 2: 83%, 3: 17%	N/A	1-2: 62%	KPS 70	100% surgery. Whole population: 20% FRT. 63% GKS. 24% chemotherapy.	EQ-5D-3 L, FACT-Br, HADS	Median 12 y after first meningioma surgery.	More anxiety and depression cf. general population. Only CNS WHO grade 2 and 3 tumours reported.	More anxiety/depression on EQ-5D. No activity problems on EQ-5D. Unremarkable emotional and functional well-being on FACT-Br. HADS score above normative sample.
Zamanipoor Najafabadi et al., 2020 [21]	Cross-sectional	>5 y follow up or following diagnosis (observational cohort). Ex: whole brain RT, NF2, neurodegenerative disease, did not speak Dutch.	190 (178 had treatment). 129 informal caregivers of meningioma patients were recruited as controls. Also compared to normative data.	N/A	63	78%	1: 148 (88%), 2: 12 (7%), unknown 8 (5%)	SB 92 (48%), convexity 93 (49%), other 5 (3%)	1-3: 109 (65%), 4-5: 40 (24%), unknown 19 (11%)	KPS 100 (90-100), cognitive deficit in 94 (49%), motor in 55 (29%)	88% surgery. 6% MRI surveillance. 5% primary RT. 14% adjuvant RT.	SF-36, QLQ-BN20, Short Form-Health and Labour Questionnaire (SF-HLQ).	Median 9 y following intervention.	Meningioma patients reported more limitations due to physical and emotional health problems compared with controls. Patients of working age less often had a paid job and reported more obstacles at work. *No grade 2 subgroup analysis.*	More emotional limitations. Increased anxiety and depression. Neurocognitive deficits in 43%. More joblessness and more perceived obstacles at work.
Wirsching et al., 2020 [28]	Cross-sectional	Histologically confirmed intracranial meningioma, >1 y follow-up	249. No comparison.	61%	56	74%	1: 219 (88%), 2 or 3: 30 (12%)	SB 89 (36%), convexity 54 (22%), falcine 35 (14%), posterior fossa. 33 (13%)	GTR (radiographic) 189 (76%), incomplete 49 (20%)	N/A	100% surgery	QLQ-C30, QLQ-BN20, MDASI-BT	>1 y postop.	One-year postop., 20% reduction in people working, 22% of full-time workers transitioned to part-time work, more patients depended on care. HRQoL improved after surgery, including headaches and seizures. Higher CNS WHO grade did not predict inferior HRQoL at 1 y. Non-responders more likely had higher CNS WHO grade.	Improved emotional and social function at 1 y postop. Cognitive function not significantly improved. Improved future uncertainty. Improved affective symptoms, symptoms interfere less with mood, activity, and daily function.
Ganefianty et al., 2020 [29]	Cross-sectional	Surgically-treated meningioma, records for 3 months to 1 y postop., who could answer questionnaires and give consent.	118. Compared to local normative population.	100%	45	80%	1: 39 (33.1%), 2: 67 (56.9%), 3: 12 (10.2%)	N/A	N/A	Postop: 36 (30.5%) independent, 70 (59.3%) mild dependence	100% surgery	EQ-5D-5 L	3-12 months postop.	HRQoL impairments across range of domains. CNS WHO grade associated with reduced HRQoL but not on multivariate analysis.	After surgery, 70% of patients reported problems with anxiety/depression and usual activities. 30.5% independent, 59.3% mild dependence, 5.9% moderate, 2.5% severe, 1.7% total.
Timmer et al., 2019 [22]	Cross-sectional	Consecutive patients >55 y.o. who underwent meningioma surgery, German or English-speaking. Ex: neuropsychological unsuitability, patient preference, unable to participate in telephone interview.	133. Compared to normative data.	N/A	67	38%	1: 109 (82%), 2: 22 (17%), 3: 2 (2%)	Convexity 14%, falx 14%, frontal 4%, tentorium cerebelli 6%, skull base 32%	1: 28%, 2: 29%, 3: 2%, 4: 7.5%, NA: 35%	90: 25%, 100: 35%	100% surgery. 0% RT.	SF-36	Mean 3.8 y postop.	Significantly lower physical function, vitality, social role functioning, mental health, and general health perception and significantly more pain between older age groups (especially 75-79) and younger patients (55-59). Most significant differences in QoL were related to co-morbidities not age. *No grade 2 or 3 analysis.*	Worse emotional role functioning and mental health in 70-74 and 75-79 groups respectively. Worse physical and physical role functioning in 75-79 and 80-84 groups respectively.
Wagner et al., 2019 [23]	Prospective	Patients scheduled for resection of newly diagnosed or recurrent meningioma, age >18 y.o.	78. No external comparison.	N/A	60	76%	1: 93%, 2: 4 (6%), 3: 1 (1%)	SB: 51%, frontal: 35%	N/A	N/A	100% surgery. 1 patient with WHO grade 1 tumour received RT.	EQ-5D-5L, SF-36	Before, 3-, and 12-months postop.	EQ-5 L scores lower at 12 months if patients had pathological PTSS-10, STAI-S, and STAI-T scores i.e. posttraumatic stress and anxiety. Non-significant increase in EQ-5L scores over follow-up period. Impaired QoL and physical disability on follow-up correlated with preoperative anxiety and depression. *No subgroup analysis for grade 2 and 3.*	Anxiety significantly decreased pre- to postop. 67.7% had abnormal anxiety scores preop, decreased to 29.6% postop. Proportion with abnormal depression scores remained stable at about 25-30%. ASI-3, STAI-S, and PTSS-10 scores decreased over course of follow-up. Role-emotional and -physical scores increased postop.
Lubgan et al., 2017 [24]	Cross-sectional	CNS WHO *grade 2 or 3* meningioma treated with adjuvant or definitive SRT.	131 (35 recurrent). No external comparison.	N/A	60 (primary), 57 (recurrent)	70% (primary), 58% (recurrent)	Primary: 2: 34%, 3: 11%, surgery, no histo.: 8%, no surgery: 47%. Recurrent: 2: 79%, 3: 13%, surgery, no histo: 8%	Primary: SB: 67%, falx: 13%, convexity: 20%. Recurrent: SB: 37%, falx: 26%, convexity: 37%	N/A	N/A	Primary: 53% surgery, 47% SRT. Recurrent: 100% surgery and SRT.	Unspecified 25 item questionnaire	42 months after treatment of primary meningiomas, 30 months for recurrent.	No significant differences in QoL after therapy of primary or recurrent meningioma.	N/A
Kim et al., 2017 [25]	Cross-sectional	Sample taken from outpatient neurosurgery clinic. Age >20 y.o., meningioma confirmed by histology, length of disease duration >1 month. Ex: metastatic brain tumour, other major health problems that could influence QoL e.g. heart or renal failure.	77. No external comparison.	N/A	53	69%	1: 70 (91%), 2: 7 (9%)	Cerebrum 53%, cerebellum 18%, others 29%	N/A	90	100% surgery. 10% adjuvant RT.	MD Anderson Symptom Inventory-Brain Tumor Module, FACT-G	Unclear.	Predictors of QoL in meningioma were KPS, cognitive symptom cluster, and physical symptom cluster. *No grade 2 subgroup analysis.*	Memory impairment most common symptom (70.1%). Four symptom clusters emerged including cognitive: common symptoms included difficult comprehension, speech, and concentration.
Henzel et al., 2013 [13]	Prospective	Meningioma, ≥18 y.o., ECOG performance ≥2, KPS ≥70%, life expectancy ≥2 y.	52. Compared to normative data.	N/A	57	75%	1: 79%; 2: 17%; 3: 5%	Falx: 8%, medial sphenoid wing: 56%, petroclival: 15%, tentorial: 6%, optic nerve sheath: 2%, olfactory: 2%	N/A	N/A	80.8% surgery. 100% SRT. Median 50.4 & 59.4 Gy for CNS WHO grade 1 & 2 tumours, respectively	SF-36	Before radiotherapy, last day of radiotherapy, then every 6 months until 24 months.	All parameters decreased compared to control but there was some recovery after 12 months. Significantly better mental component scores with previous operations. *CNS WHO grade 2 and 3 tumours not reported separately.*	Patients had better mental component scale than physical. Physical role functioning and emotional role functioning declined the most, followed by social and physical functioning with RT. All increased following RT completion and peaked at 6 months. Physical functioning, physical role functioning, social role functioning, emotional role functioning worse in pre-op population vs. normative pop.
Jakola et al., 2012 [14]	Prospective	Histologically confirmed meningioma, age ≥18 y.o. Ex: recurrent meningioma.	46. No external comparison.	N/A	55	67%	1: 83%, 2: 17%	Convexity: 24%, parasagittal or falx: 33%, supratentorial skull base: 35%, infratentorial: 8%	1-2: 66%, 3: 17%, 4-5: 17%	KPS 85±11	100% surgery. 7% had later GKS, 2% had conventional RT postop.	EQ-5D-3L	1-3 days before surgery; 6 weeks postop. (short-term); 10-58 months postop. (long-term).	Surgery reduced pain/discomfort, anxiety/depression, improved capability of performing usual activities. Clinically significant improvement at long-term assessment in 25 patients (49%), a significant deterioration was reported in 10 (20%). *CNS WHO grade 2 and 3 tumours not reported separately.*	Surgery reduced anxiety/depression and improved performance of usual activities. Of those worse after surgery, 2 had reduced mobility, 4 had reduction of usual activities, four had more anxiety/depression.
Miao et al., 2010 [26]	Prospective	Histologically confirmed meningioma and operated.	Men.: 147, age-matched ctrl.: 96	N/A	Men.43, ctrl. 42	Men. 59%, ctrl. 67%	1: 80%, 2: 7%, 3: 6%	Convexity: 39%, parasagittal: 3%, falx: 16%, olfactory groove: 9%, sphenoid ridge: 13%, clivus: 5%, intraventricular: 6%, cerebellum: 4%	0: 8%, 1: 18%, 2: 20%, 3: 27%, 4: 27%	N/A	100% surgery. 0% RT.	Chinese questionnaire based on WHOQOL-100	Before and after surgery, not further specified.	QoL lower in meningioma patients than controls, postop QoL better than preoperative. CNS WHO grade was a significant predictor of lower QoL.	Psychological dimension only one not to improve after surgery.

N/A, not assessed or not reported; cf., compared with; QoL, quality-of-life; RT, radiotherapy; WHOQOL-100, World Health Organisation Quality of Life-100 Scale; Men., meningioma; crtl., control; SRT, stereotactic radiotherapy; y, year(s); y.o., years-old; postop, postoperative

One study had age-matched controls [26], another used caregivers of meningioma patients as controls [21], eight studies compared their results to normative results in the general population [8, 13, 15, 19, 20, 22, 27, 29], and five studies did not have control groups [14, 23–25, 28]. Surgery was the primary treatment modality in all studies. Radiotherapy of some sort was employed for different proportions of patients in most other studies [8, 13–15, 19–21, 24, 25]. Two studies compared HRQoL in conservatively and operatively managed meningiomas [8, 21].

HRQoL tools included EORTC QLQ-C30 [8, 19, 20, 28], QLQ-BN20 [8, 19–21, 28], RAND SF-36 [8, 13, 21–23], EQ-5D-5L [23, 29], EQ-5D-3L [14, 15], FACT-Br [15, 27], FACT-G [25, 27], SF-HLQ [21], MDASI-BT [25], and a Chinese questionnaire based on WHOQOL-100 [26]. One study used an unspecified 25-item questionnaire [24]. HRQoL questionnaires were completed a mean of 6.4 years following treatment for studies reporting average follow-up time and were also measured earlier in treatment courses in a number of studies [13, 14, 19, 23].

Data from included studies are summarised in Table 1. Significant and clinically relevant results from the original studies are reported here.

### HRQoL of CNS WHO grade 2 and 3 meningioma versus grade 1

Only four included studies recruited all WHO grades of meningioma and reported on the impact of WHO grade in HRQoL. Both Wirsching et al. and Kofoed Lauridsen et al. found that higher WHO grade did not predict inferior HRQoL at 1 year and 7 years postoperatively, respectively, although the latter only included subfrontal meningiomas, while the former adjusted for meningioma location [27, 28]. Ganefianty et al. reported that WHO grade was associated with worse HRQoL on univariate but not multivariate analysis with 57% of their included patients harbouring WHO grade 2 meningioma but meningioma location was not reported [29]. Miao et al. demonstrated that WHO grade was a significant predictor of worse HRQoL on multivariate analysis that also adjusted for meningioma location [26].

### HRQoL of CNS WHO grade 2 and 3 meningioma versus normative data and healthy controls

Clinically relevant HRQoL impairments across several domains were reported by most studies that compared their results to normative populations [8, 13, 20–22, 26, 29]. However, these studies did not report standalone comparisons between WHO grade 2 and 3 meningioma and normative populations. One study of WHO grade 2 and 3 meningioma compared their results to normative data and their main outcome was a specifically increased rate of anxiety and depression in meningioma [15]. Other results from this study included a lower proportion of meningioma patients reporting ‘full health’– 15% vs. 44% in the general population. Otherwise, meningioma patients that answered the surveys (25.5% of total study population) reported comparable HRQoL to the general population [15].

### Psychological health, employment status, and other patient-reported outcomes

Most included studies reported decreased psychological health in meningioma patients relative to controls or normative data. One study of HRQoL in WHO grade 2 and 3 meningioma did not report on psychological or role functioning outcomes [24]. The other study of exclusively WHO grade 2 and 3 meningioma reported an increase in symptoms of anxiety and depression compared to the general population a median of 12 years postoperatively [15]. A study in which 67% of patients had either WHO grade 2 or 3 meningioma found that after surgery, 70% of patients reported problems with anxiety or depression and usual activities [29]. This study also found that 31% of patients were independent, 59% had mild dependence, and 6%, 3%, and 2% had moderate, severe, and total dependence up to 1 year postoperatively, respectively [29]. One study that included a small proportion (~ 8%) of WHO grade 2 subfrontal meningioma found that patients had less anxiety and depression than reference populations a mean of 7.1 years postoperatively [27].

Of the other included studies, some found that mental health, including anxiety and depressive symptoms were improved with treatment [13, 14, 19, 23, 28], while others found that emotional wellbeing was still worse than reference populations after treatment [8, 21, 26], or worse for certain age groups [22]. Depressive symptoms were less likely to change postoperatively than anxiety [23]. Cognitive symptoms were prominent and less likely to improve [8, 19, 20, 25, 28]. There was no clear trend for role functioning with more studies demonstrating deficits [8, 20, 21, 28], some demonstrating improvements [23, 28], one study showing a decrease with radiotherapy followed by normalisation back to baseline [13], and another demonstrating no change after proton therapy [19].

### HRQoL in meningioma patients before and after intervention (surgery)

Four studies reported an improvement in HRQoL after surgery for meningioma; three had measured HRQoL pre-operatively [14, 26, 28], and in another study improvement was implied and better than the general population [27]. Of these four studies, WHO grade was unrelated to HRQoL in two studies [27, 28], negative in another [26], and not evaluated in the other [14]. Three studies reported a decrease in HRQoL following surgery, especially following re-resection [21], although none had measured HRQoL pre-operatively [21, 22, 29]. Increased WHO grade was associated with reduced HRQoL in one of these studies on univariate, but not multivariate analysis [29]. Four studies reported no change in HRQoL following surgery [8, 15, 23, 24], although only two studies measured HRQoL pre-operatively [23], or had a non-operative cohort [8]. Two studies investigated WHO grade 2 and 3 meningioma only but did not measure HRQoL pre-operatively [15, 24].

### HRQoL in meningioma patients before and after intervention (radiotherapy)

In one study that looked at meningioma patients managed with radiation, either primary, adjuvant, or salvage, global health status was rated lower than normative populations with decreased physical, cognitive, and social function, with corresponding increases in fatigue, pain, and other symptoms [20]. Another study investigated HRQoL before and after stereotactic radiotherapy, given to all patients, and found that there was a decline in mean values of HRQoL parameters after treatment, down from a reduced baseline relative to a normative population, but normalised towards their initial (reduced) values after 12 months [13]. The study population included WHO grade 2 and 3 tumours (combined 22% of total) but these higher grade tumours were not reported separately [13]. Proton beam therapy did not have a negative impact on HRQoL in the only study investigating the intervention, but did not report on WHO grade 2 and 3 meningioma specifically [19].

### Assessment of reporting level of HRQoL data in included articles

Reporting level of HRQoL data from included studies is summarised in Table 2. Mean and median reporting level scores were 14 (range 6–17) and 13 articles had HRQoL data that was deemed sufficiently reported (≥ 13 points, Table 2). All articles described the generalisability of their HRQoL findings and most included a copy or reference of the instrument used (93%), described their HRQoL outcome in the title or abstract (87%), stated their HRQoL hypothesis in the introduction (87%), described raw HRQoL data (87%), and included adequate interpretation of their HRQoL findings (87%). Most articles did not report on HRQoL methodology and statistics (20% and 27%). On other criteria, 60–80% of articles scored the highest possible score.

**Table 2:** Assessment of HRQoL reporting levels of included studies

Author (year)	Title & abstract (1 pnt)	Introduction, background & objectives (1 pnt)	Methods (6 pnt)		Results (3 pnt)			Discussion (4 pnt)			Protocol/copy of instrument (1 pnt)	Total points (max 18)
			Outcomes (6 pnt)	Statistical methods (2 pnt)	Participant flow/missing data (1 pnt)	Baseline data (1 pnt)	Outcomes and estimation (1 pnt)	Limitations (1 pnt)	Generalisability (1 pnt)	Interpretation (2 pnt)		
Krcek et al., 2023 [19]*	1	1	3	1	1	1	1	1	1	1	1	13
Kofoed Lauridsen et al., 2023 [27]*	1	1	5	1	1	0	1	1	1	2	1	15
Keshwara et al., 2023 [8]*	1	1	6	2	1	0	1	1	1	2	1	17
Lisowski et al., 2022 [20]*	1	1	3	2	1	1	1	1	1	2	1	15
Pettersson-Segerlind et al., 2022 [15]*	1	1	4	1	0	1	1	1	1	2	1	14
Zamanipoor Najafabadi et al., 2020 [21]*	0	1	4	2	1	0	1	1	1	2	1	14
Wirsching et al., 2020 [28]*	1	1	5	1	1	1	0	1	1	2	1	15
Ganefianty et al., 2020 [29]	1	1	3	1	0	0	1	0	1	2	1	11
Timmer et al., 2019 [22]*	1	1	5	1	0	1	1	1	1	2	1	15
Wagner et al., 2019 [23]*	1	1	4	1	0	1	1	1	1	2	1	14
Lubgan et al., 2017 [24]	0	1	1	1	0	0	0	0	1	2	0	6
Kim et al., 2017 [25]*	1	1	6	1	1	1	1	1	1	2	1	17
Henzel et al., 2013 [13]*	1	0	4	0	1	1	1	1	1	2	1	13
Jakola et al., 2012 [14]*	1	0	6	2	1	1	1	1	1	1	1	16
Miao et al., 2010 [26]*	1	1	4	1	0	1	1	0	1	2	1	13
% of studies scoring maximum score per criterion	87%	87%	20%	27%	60%	67%	87%	80%	100%	87%	93%	Mean 14

* Articles with sufficient reporting level (predefined cutoff ≥ 13 points)

## Discussion

While meningioma have generally favourable prognoses among primary CNS tumours, WHO grade 2 and 3 meningioma have worse outcomes with recurrence rates ranging from 30 to 50% and 50 to 94% [30–33], respectively. Less is known about HRQoL in WHO grade 2 and 3 meningioma. The present systematic review identified reduced HRQoL in most of the 15 studies reported here that included WHO grade 2 and 3 meningioma, although only two studies exclusively reported on WHO grade 2 and 3 meningioma [15, 24], and four further studies considered WHO grade in statistical analysis [26–29]. Of the 13 studies reporting on all WHO grades, 17% and 6% of patients were grade 2 and 3, respectively. QLQ-C30, QLQ-BN20, SF-36, and EQ-5D questionnaires were the most common HRQoL surveys used.

WHO grade 2 and 3 meningioma were associated with reduced HRQoL in two studies that reported direct comparison and no difference in another two. Psychological domains were reduced in most studies compared to normative data or controls including in one of the two studies reporting on WHO grade 2 and 3 tumours exclusively [15]. Mental health appeared to improve with treatment in some studies but not all and depressive symptoms improved less than anxiety symptoms. Cognitive symptoms were common and generally persisted after treatment. Surgery was associated with improved, reduced, and unchanged HRQoL in near equal proportions of studies and radiotherapy was associated with unchanged or reduced HRQoL. Study outcomes could not be pooled due to heterogenous patient cohorts, treatment regimens, and HRQoL questionnaires. The level of HRQoL reporting of most articles was of good quality although methodology was uniformly poorly reported and only three survey tools have been validated for use in meningioma patients including the FACT-G/FACT-BR [34], and a study-specific questionnaire used by Miao et al. [35].

Reduced HRQoL in meningioma is related to symptoms of raised intracranial pressure (including headaches, visual changes), neurological deficit (including weakness, visual loss, speech loss), seizures, and endocrine dysfunction in sellar region meningiomas [36]. Many of these symptoms and signs are more prominent in WHO grade 2 and 3 meningiomas which grow faster, become larger, and often invade the brain [37].

Improved HRQoL following surgery for meningioma could be explained by the reversal of mass effect causing raised intracranial pressure, its associated symptoms, and neurological deficits. Seizures can also improve after meningioma surgery [38]. Few of the studies included in this review attempted to build multivariate models to control for symptoms and neurological deficits that could be contributing to the reduction in HRQoL.

Two studies evaluated both operatively and non-operatively managed meningiomas and observed no significant difference in HRQoL between the groups [8, 24]. While this appears in contrast to the results of studies that demonstrated improved HRQoL with surgery, different patient selection or selection bias probably accounts for these differences. Meningioma that are observed are more likely to be small, slow growing, and not causing symptoms, which likely confers more favourable HRQoL [4].

Radiotherapy is controversial in WHO grade 2 meningioma but commonly offered in grade 3 meningioma [4]. Radiation-associated symptoms or ‘toxicity’ are problematic for patients with CNS tumours and form part of the risk-benefit decision making process for deciding whether to offer adjuvant radiotherapy or use it as salvage therapy for recurrent or progressive disease [39]. Institutions represented by the included studies report different standards of care regarding adjuvant radiotherapy, which has resulted in different proportions of patients receiving radiotherapy and making comparison and synthesis of these studies more difficult.

A systematic review of HRQoL in meningioma that focussed largely on WHO grade 1 tumours was published in 2016 [17]. Three studies included in the present systematic review were also featured in the 2016 review [13, 14, 26], as the only three studies including WHO grade 2 or 3 meningioma. The authors reached similar conclusions to the present review, specifically that meningioma patients generally report lower HRQoL than healthy controls, HRQoL after radiotherapy was comparable to pre-treatment HRQoL, but interestingly, long-term follow-up showed persistent reduced HRQoL relative to healthy controls. The authors graded their included studies’ patient-reported outcome reporting lower than in the present review, perhaps reflecting the different grading tools used. Another more recent review looked at the effect of WHO grade in three studies, two of which were included in the present review, and found a significant negative relationship with HRQoL in one study and no association in the other two studies [40]. While the present review focussed on WHO grade 2 and 3 meningioma, all but two included studies also reported on WHO grade 1 meningioma, which always outnumbered WHO grade 2 and 3 meningioma in each study population, due to the relative incidence of each WHO grade. A key finding of the present review is that HRQoL in WHO grade 2 and 3 meningioma is reported rarely. Even if WHO grade 2 and 3 meningiomas are included in studies reporting on HRQoL, they are very infrequently reported in subgroup analysis. The literature would be greatly enhanced by more of these studies reporting WHO grade 2 and 3 subgroup analysis.

HRQoL in patients with low- and high-grade glioma have been evaluated in similar studies [25], although the significant difference in survival between grades of glioma makes comparison between grades more difficult. Low-grade glioma itself has been shown to confer reduced HRQoL, especially in cognitive functioning, fatigue, and in association with seizures [41]. HRQoL is even worse in high-grade glioma [42]. Cognitive, fatigue, and seizure-related effects on HRQoL could be considered consequences of the intra-axial nature of glioma, symptoms that are less common in meningioma [43], although few studies have compared HRQoL in meningioma with glioma [43].

### Limitations

The present systematic review is limited by the heterogeneity of patient groups (primary, recurrent), inclusion and exclusion criteria, interventions (surgery, radiotherapy, other adjuvant therapy), timing of HRQoL measurement, and questionnaire differences. In the absence of high quality, unbiased data collected in a randomised controlled trial, it is difficult to compare HRQoL between treatment groups, e.g. surgery versus radiotherapy, and control for confounding factors such as meningioma location and WHO grade. Furthermore, many included studies were limited by low response rates to questionnaires, which may represent a selection bias for groups of patients with or without certain symptoms including cognitive deficit.

There are two specific limitations in the ability to extract data on WHO grade 2 and 3 meningioma outcomes. Firstly, the small proportion of patients in these cohorts reduces the power of studies to associate higher grade meningioma with specific outcomes. Secondly, WHO grade 2 and 3 meningioma have a different management paradigm which more commonly employs radiotherapy, which is poorly captured and heterogenous in reported series.

### Implications for practice and research

The present study has demonstrated that patients with WHO grade 2 and 3 meningioma have reduced HRQoL relative to controls, in some cases many years after treatment. While the evidence base is small and heterogenous, this information highlights the need for services to remain vigilant of patients’ long-term outcomes and care provision, even after patients have been discharged from neurosurgical services.

Future studies of HRQoL in WHO grade 2 and 3 meningioma should involve the prospective recruitment of patients and completion of both standardised questionnaires comparable to large normative populations (QLQ-C30, QLQ-BN20, SF-36) and meningioma-specific tools (MQoL [44], FACT-MNG [45]) that can account for the location-associated deficits of certain meningioma, e.g. vision, speech, and olfaction. This information would aid in assessing the individual impacts of WHO grade and meningioma location on HRQoL. A core outcome data set for meningioma may help guide selection of appropriate tools [46].

## Conclusions

The present systematic review summarises 15 studies reporting on HRQoL in patients with WHO grade 2 and 3 meningioma. Most questionnaires used have not yet been validated in meningioma patients, although many have been validated in other types of brain tumours with worse prognoses. Reporting quality of HRQoL outcomes was generally good although most studies did not adequately report on their methodology. Results of the included studies are mixed as to the effect of WHO grade on HRQoL but suggest worse HRQoL outcomes with higher WHO grades. These conclusions are limited by the small number of studies exclusively recruiting or analysing WHO grade 2 and 3 meningioma and future work in this area would benefit from larger prospective studies of more patients with grade 2 and 3 meningioma with validated meningioma-specific HRQoL tools.

## Electronic supplementary material

Below is the link to the electronic supplementary material.

Supplementary Material 1

## Data Availability

The literature datasets generated during and/or analysed during the current study are available from the corresponding author on reasonable request.
