# Supplementary material for: Health-related quality-of-life outcomes in CNS WHO grade 2 and 3 meningioma: a systematic review
Source: Neurosurg Rev. 2025 Feb 27;48(1):268. doi: 10.1007/s10143-025-03420-5 (PMC11865157; doi:10.1007/s10143-025-03420-5)
Supplement: Supplementary file 1 — Supplementary Material 1 [file 10143_2025_3420_MOESM1_ESM.docx]

**Supplementary Materials**

**Appendix 1** Ovid MEDLINE search

Ovid MEDLINE(R) <1946 to May Week 3 2023>

1 (atypical adj6 meningioma*).mp. [mp=title, book title, abstract, original title, name of substance word, subject heading word, floating sub-heading word, keyword heading word, organism supplementary concept word, protocol supplementary concept word, rare disease supplementary concept word, unique identifier, synonyms, population supplementary concept word, anatomy supplementary concept word] 1136

2 (malignant adj6 meningioma*).mp. [mp=title, book title, abstract, original title, name of substance word, subject heading word, floating sub-heading word, keyword heading word, organism supplementary concept word, protocol supplementary concept word, rare disease supplementary concept word, unique identifier, synonyms, population supplementary concept word, anatomy supplementary concept word] 1168

3 (grade adj2 II adj6 meningioma*).mp. [mp=title, book title, abstract, original title, name of substance word, subject heading word, floating sub-heading word, keyword heading word, organism supplementary concept word, protocol supplementary concept word, rare disease supplementary concept word, unique identifier, synonyms, population supplementary concept word, anatomy supplementary concept word] 583

4 (grade adj2 III adj6 meningioma*).mp. [mp=title, book title, abstract, original title, name of substance word, subject heading word, floating sub-heading word, keyword heading word, organism supplementary concept word, protocol supplementary concept word, rare disease supplementary concept word, unique identifier, synonyms, population supplementary concept word, anatomy supplementary concept word] 334

5 (grade adj2 "2" adj6 meningioma*).mp. 120

6 (grade adj2 "3" adj6 meningioma*).mp. [mp=title, book title, abstract, original title, name of substance word, subject heading word, floating sub-heading word, keyword heading word, organism supplementary concept word, protocol supplementary concept word, rare disease supplementary concept word, unique identifier, synonyms, population supplementary concept word, anatomy supplementary concept word] 55

7 exp Specialties, Surgical/ 218776

8 surg*.mp. [mp=title, book title, abstract, original title, name of substance word, subject heading word, floating sub-heading word, keyword heading word, organism supplementary concept word, protocol supplementary concept word, rare disease supplementary concept word, unique identifier, synonyms, population supplementary concept word, anatomy supplementary concept word] 3307403

9 neurosurg*.mp. [mp=title, book title, abstract, original title, name of substance word, subject heading word, floating sub-heading word, keyword heading word, organism supplementary concept word, protocol supplementary concept word, rare disease supplementary concept word, unique identifier, synonyms, population supplementary concept word, anatomy supplementary concept word] 83109

10 exp Radiotherapy/ 206643

11 radiotherap*.mp. [mp=title, book title, abstract, original title, name of substance word, subject heading word, floating sub-heading word, keyword heading word, organism supplementary concept word, protocol supplementary concept word, rare disease supplementary concept word, unique identifier, synonyms, population supplementary concept word, anatomy supplementary concept word] 342743

12 radiosurg*.mp. [mp=title, book title, abstract, original title, name of substance word, subject heading word, floating sub-heading word, keyword heading word, organism supplementary concept word, protocol supplementary concept word, rare disease supplementary concept word, unique identifier, synonyms, population supplementary concept word, anatomy supplementary concept word] 22720

13 Radiation therap*.mp. [mp=title, book title, abstract, original title, name of substance word, subject heading word, floating sub-heading word, keyword heading word, organism supplementary concept word, protocol supplementary concept word, rare disease supplementary concept word, unique identifier, synonyms, population supplementary concept word, anatomy supplementary concept word] 78696

14 exp Drug Therapy/ 1504164

15 chemotherap*.mp. [mp=title, book title, abstract, original title, name of substance word, subject heading word, floating sub-heading word, keyword heading word, organism supplementary concept word, protocol supplementary concept word, rare disease supplementary concept word, unique identifier, synonyms, population supplementary concept word, anatomy supplementary concept word] 509484

16 1 or 2 or 3 or 4 or 5 or 6 2499

17 8 or 9 or 10 or 11 or 12 or 13 or 14 or 15 5036580

18 16 and 17 1479

| **Supplementary Table 1** Patient-reported outcome (PRO) measure reporting standards adapted from Brundage et al., 2013 [16] | | |
| --- | --- | --- |
| Reporting standard category | Points available | Criteria |
| Title & abstract | 1 | The title of the paper should be explicit as to the RCT including a PRO |
| Intro, background, objectives | 1 | The PRO hypothesis should be stated and should specify the relevant PRO domain(s) if applicable |
| Methods: outcomes | 6 | 1. The mode of administration of the PRO tool and the methods of collecting data (e.g., telephone, other) should be described 2. The rationale for choice of the PRO instrument used should be provided 3. Evidence of PRO instrument validity and reliability should be provided or cited 4. The intended HRQL data collection schedule should be provided 5. PROs should be identified in the trial protocol; post hoc analyses should be identified 6. The status of PRO as either a primary or secondary outcome should be stated |
| Methods: statistics | 2 | 1. There should be evidence of appropriate statistical analysis and tests of statistical significance for each PRO hypothesis tested 2. Statistical approaches for dealing with missing data should be explicitly stated, and the extent of missing data should be stated |
| Results: participant flow | 1 | A flow diagram or a description of the allocation of participants and those lost to follow-up should be provided for PROs specifically |
| Results: Baseline data | 1 | The study patients’ characteristics should be described, including baseline PRO scores |
| Results: Outcomes & estimation | 1 | For each primary and secondary outcome, results for each group, and the estimated effect size and its precision (such as 95 % confidence interval) |
| Discussion: limitations | 1 | The limitations of the PRO components of the trial should be explicitly discussed |
| Discussion: generalisability | 1 | Generalizability issues uniquely related to the PRO results should be discussed, if applicable |
| Discussion: interpretation | 2 | 1. The clinical significance of the PRO findings should be discussed 2. The PRO results should be discussed in the context of the other clinical trial outcomes |
| Other: protocol | 1 | A copy of the instrument should be included if it has not been published previously |
| Total score | 18 |  |
